# Editorial: Can Chinese medicines affect diarrhea via effects of the intestinal microbiota on the renal-intestinal axis?

**DOI:** 10.3389/fcimb.2025.1593758

**Published:** 2025-05-13

**Authors:** Xinhua Shu, Nenqun Xiao, Xue-Wei Ye, Maijiao Peng

**Affiliations:** ^1^ Pu Ai Medical School, Shaoyang University, Shaoyang, Hunan, China; ^2^ Department of Biological and Biomedical Sciences, Glasgow Caledonian University, Glasgow, United Kingdom; ^3^ College of Pharmacy, Hunan University of Chinese Medicine, Changsha, Hunan, China; ^4^ Department of Basic Medical Sciences, Shulan International Medical College, Key Laboratory of Pollution Exposure and Health Intervention of Zhejiang Province, Zhejiang Shuren University, Hangzhou, China

**Keywords:** diarrhea, intestinal microbiota, renal-intestinal axis, Chinese medicines, treatment

Diarrhea, characterized by at least three loos or watery stool per day, is a common heath issue and primarily affects infants and young children, accounting for over 440,000 deaths of children under five years of age each year worldwide (https://www.who.int/news-room/fact-sheets/detail/diarrhoeal-disease ). Diarrhea is classified into acute diarrhea (lasting less than 14 days) and chronic diarrhea (lasting over 14 days). The most common causes of acute diarrhea are bacterial, viral and parasitical infections (Abad and Safdar, 2021; Li et al., 2021; Meisenheimer et al., 2022); some drugs (e.g. clarithromycin) and toxins (e.g. sea food poisoning) can also cause acute diarrhea (Abad and Safdar, 2021; Wang et al., 2024). Chronic medical conditions that affect the intestine, such as inflammatory bowel disease and irritable bowel syndrome, are the common cause of chronic diarrhea (Chu et al., 2020). Treatment of diarrhea is mainly through the supply of fluid and salt via oral or (in severe cases) intravenous rehydration therapy. Alternative treatments include antidiarrhea agents, zinc supplementation and nutrient-rich foods (https://www.who.int/news-room/fact-sheets/detail/diarrhoeal-disease; Meisenheimer et al., 2022).

The gastrointestinal tract contains trillions of microorganisms (termed gut microbiota) including bacteria, viruses, fungi, archaea and protists. Gut microbiota are involved in multiple functions, such as food digestion and uptake, synthesis of micronutrients, maintenance of gut barrier integrity, and regulation of host immune responses (Adak and Khan, 2019). Numerous factors e.g. diet, inflammation, infection, and the host’s genetic background and immediate environment can affect the composition and function of gut microbiota (Lynch and Pedersen, 2016; Adak and Khan, 2019). Imbalance of gut microbiota, termed dysbiosis and characterized by enriched harmful microorganisms, reduced richness of beneficial microorganisms and loss of overall microbial diversity, has been associated with various types of disease, including inflammatory, metabolic and neurological disorders (Lynch and Pedersen, 2016). Diarrhea is associated with gut dysbiosis, as pathogenic microorganisms suppress the growth of beneficial microorganism and produce toxic substances, resulting in dysregulation of gut function and immune response, which contribute to diarrhea. Bacterial infection is the most common cause of diarrhea in developing countries (Li et al., 2021).

Traditional Chinese medicine (TCM) views diarrhea as the consequence of spleen deficiency associated with stomach, liver and kidney, characterized by six syndromes: spleen and stomach deficiency syndrome; intestinal damp-heat syndrome; kidney-yang deficiency; liver-qi stagnation with spleen deficiency; retention of food in the stomach syndrome; and stagnation of cold-damp syndrome (Zhang et al., 2017). TCM has been used to treat diarrhea for thousands of years, with oral administration of TCM is supposed to regulate gut microbiota homeostasis. However, the underlying protective mechanisms are not fully understood. In the current Research Topic, we collect reviews and research papers to advance the understanding of TCM in treatment of diarrhea via the mediation of gut microbiota homeostasis.

Based on the TCM classification of diarrhea syndromes, Yu et al. examined the effects of diarrhea with spleen deficiency and dampness syndrome on gastrointestinal function, fluid and energy metabolism, and gut microbiota. The authors found that diarrhea of this type caused gastrointestinal dysfunction, disruption of fluid and energy metabolism, and alternation of composition and diversity of gut microbiota in mice (Yu et al.). Zhou et al. reviewed the progress of TCM in treating diarrhea via modulation of the gut-kidney axis. There is a functional link between gut microbiota and the kidney. Gut microbes can synthesize metabolites such as short chain fatty acids (SCFAs) that are of benefit to the kidney; some gut microbe-derive metabolites, such as p-cresyl sulfate, can exacerbate kidney disease progression. It is well known that diarrhea can cause kidney injury due to both reduced blood flow to the kidney and imbalanced electrolyte as a consequence of dehydration; kidney injury, in turn, can cause diarrhea. The protective effect of TCM against kidney disease-associated diarrhea is through regulating gut microbiota homeostasis, maintaining intestinal barrier integrity, reducing inflammation and enhancing excretion of toxic microbe-derived metabolites (Zhou et al.). Yu et al. also reviewed the biological significance of diarrhea treatment with TCM through the promotion of urination and regulation of bowel movements. The authors discussed the microbial-derived metabolites and the renal-intestinal axis, particularly how individual metabolites affect renal function and fluid metabolism. Additionally, the authors summarized that TCM’s therapeutic effects against diarrhea are through the improvement of gut microbiota homeostasis and the production of more beneficial metabolites that function as intermediary factors that enhance urination and fluid metabolism and relieve diarrhea (Yu et al.). Shao et al. investigated the therapeutic effect of TCM, Tongxieyaofang (TXYF), on diarrhea with liver stagnation and spleen deficiency syndrome. TXYF contains four Chinese medicinal herbs that network pharmacology has identified as containing 32 bioactive compounds and having 145 potential target genes. There are 94 intersection targets between compounds of TXYF and targets associated with diarrhea. These intersection targets are involved primarily in inflammation pathways. TXYF administration altered the diversity and composition of gut microbiota and regulated gut microbial function in mice with diarrhea (Shao et al.).

Inflammatory bowel disease (IBD) is a chronic disorder, predominantly affecting the gastrointestinal tract (Agrawal et al., 2022). IBD is classified mainly into ulcerative colitis and Crohn’s disease, both of which may involve abdominal pain, diarrhea and other symptoms (Ancona et al., 2021). In a review paper, Lu et al. discussed the involvement of gut microbiota and CD4^+^T cells in the pathogenesis of IBD. Studies in IBD animal models have demonstrated that gut dysbiosis is associated with IBD, possibly via decreasing production of beneficial metabolites, such as SCFAs and bile acids, which can regulate CD4^+^T cell differentiation. The therapeutic effects of TCM treatment in IBD animal models or IBD patients are possibly through modulating gut microbiota and CD4^+^T cell differentiation/activation and suppressing intestinal inflammation, so reversing IBD syndrome (Lu et al.). Pang et al. assessed the therapeutic effect of *Saussurea costus* (SC), a TCM herb against ulcerative colitis. The authors identified 291 compounds from SC water extract using high-performance liquid (HPLC)/mass spectrometry (ms). SC administration alleviated pathology and modulated gut microbiota in colitis mice. The results of this study suggest that SC has therapeutic potential for patients with ulcerative colitis. Irritable bowel syndrome (IBS) is a functional gastrointestinal disease, caused mainly by a defect in communication between the brain and the gut (Ancona et al., 2021). Diarrhea is a common symptom of IBS. Although the pathophysiology of IBS is not fully understood, dysbiosis of gut microbiota is associated with its onset. Deng et al. examined the pathological features and gut microbiota in IBS mice compared to control animals. The authors reported that IBS mice showed emotional hyper-reactivity and irritability, increased inflammation, and decreased diversity and function of microbiota (Deng et al.). Additionally, treatment with Qiwei Baizhu powder, a TCM that has been already used to treat IBS, resulted in increased diversity of mucosa-associated microbiota, decreased serum levels of proinflammatory cytokines and alleviation of diarrhea symptoms in mice with antibiotic-induced diarrhea, possibly via mediation of the inflammation pathways (Zhang et al.).

This Research Topic has also collected articles associated with diarrhea. Constipation is a common gastrointestinal complication, characterized by reduced frequency of stool passage (Schiller, 2019). Spleen deficiency can cause constipation due to dysregulation of fluid metabolism in the body. TCM has been widely used to treat constipation. Here, both Guo et al. and Liang et al. explore the therapeutic potential and underlying mechanisms of Massa Medicata Fermentata (MMF) against spleen deficiency constipation. MMF is a fermented functional food and can be used as a TCM in treatment of digestive disorders. MMF regulated the diversity and composition of gut microbiota, activity of intestinal enzymes, and the serum levels of short peptides, monosaccharide and neurotransmitters in mice with spleen deficiency constipation, so contributing to the reversal of constipation syndromes (Guo et al.; Liang et al.). Another TCM, Zhishi Daozhi decoction (ZDD), which contains eight herbs, also demonstrated therapeutic effect against constipation. Similar to that of MMF treatment, ZDD administration regulated gut microbiota and serum levels of peptides and neurotransmitter and ameliorated constipation syndromes (Fang et al.). Gastrointestinal diseases, such as IBD, are often associated with osteoporosis, characterized by a decrease in bone density and alternation of bone microarchitecture (Oh et al., 2018). Gut dysbiosis also plays an important role in the pathogenesis of osteoporosis (Zhang et al., 2024). Li et al. showed that Zhuanggu Shubi ointment, a TCM containing 10 herbs, increased bone density, improved the integrity of the intestinal barrier structure, inhibited intestinal inflammation and regulated gut microbiota, resulting in alleviation of osteoporosis syndromes in a rat postmenopausal osteoporosis model. The studies discussed here are targeted to disease models and patients; in fact, Li et al. demonstrated that Tanreqing injection, a TCM consisting of water extract from five medicinal herbs together with bioactive constituents, showed non-significant effects on diversity, composition and function of gut microbiota.

In summary, the collected 13 articles will shed new insights on the therapeutic effect and the underlying molecular functional mechanisms of TCM in treating diarrhea. TCM regulates gut microbiota homeostasis and alleviate diarrhea or diarrhea-associated syndromes via gut-kidney or gut-brain axis. Since TCM normally contains numerous compounds and is thought to be involved in multiple functional pathways, further studies are required to elucidate the functional role of individual or combinations of compounds in treating diarrhea.
